# Intersecting systemic and personal barriers to accessing social services: qualitative interviews in northern California

**DOI:** 10.1186/s12889-021-11981-5

**Published:** 2021-10-24

**Authors:** Hilary Placzek, Stephanie Cruz, Michelle Chapdelaine, Mary Carl, Sara Levin, Clarissa Hsu

**Affiliations:** 1 Health Leads, San Francisco, CA USA; 2 Ariadne Labs, Boston, MA USA; 3 Ontrak, Inc., San Francisco, CA USA; 4 Kaiser Permanente Washington Health Research Institute, Seattle, WA USA; 5 Department of Anthropology, University of Washington, Seattle, WA USA; 6 Contra Costa Public Health Clinical Services, Martinez, CA USA

**Keywords:** Social determinants of health, Qualitative patient-reported interviews, Clinical navigation services for addressing social needs, Health and social service systems, Population health

## Abstract

**Background:**

Addressing social risks in the clinical setting can increase patient confidence in the availability of community resources and may contribute to the development of a therapeutic alliance which has been correlated with treatment adherence and improved quality of life in mental health contexts. It is not well understood what barriers patients face when trying to connect to community resources that help address social risks. This paper aims to describe patient-reported barriers to accessing and using social needs-related resources to which they are referred by a program embedded in a safety net primary care clinic.

**Methods:**

This is a qualitative assessment of patient-reported barriers to accessing and using social needs assistance programs. We conducted over 100 in-depth interviews with individuals in Northern California who participated in a navigation and referral program to help address their social needs and describe a unique framework for understanding how policies and systems intersect with an individual’s personal life circumstances.

**Results:**

Individuals described two distinct domains of barriers: 1) systems-level barriers that were linked to the inequitable distribution of and access to resources, and 2) personal-level barriers that focused on unique limitations experienced by each patient and impacted the way that they accessed services in their communities. While these barriers often overlapped or manifested in similar outcomes, this distinction was key because the systems barriers were not things that individuals could control or overcome through their own initiative or by increasing individual capacity.

**Conclusions:**

Respondents describe intersecting systemic and personal barriers that compound patients’ challenges to getting their social needs met; this includes both a picture of the inequitable distribution of and access to social services and a profile of the limitations created by individual life histories. These results speak to the need for structural changes to improve adequacy, availability, and accessibility of social needs resources. These findings highlight the need for advocacy to address systems barriers, especially the stigma that is faced by people who struggle with a variety of health and social issues, and investment in incentives to strengthen relationships between health care settings and social service agencies.

## Background

Attending to the upstream and downstream impacts of social determinants of health in clinical settings is a critical component of improving the overall health and well-being of patients [1]. Most recently, the global COVID-19 pandemic has further highlighted how social risks like housing instability, food instability, and access to healthcare impact exposure, testing, severity, and adequate treatment for coronavirus infection [2]. An assessment of programs designed to identify and support patients with unaddressed social needs have found that addressing social needs can lead to clinically meaningful improvements in outcomes like blood pressure and LDL-cholesterol [3]. More importantly, studies have found that addressing these social drivers of health in the clinical setting can increase patient confidence in the availability of community resources [3] and may contribute to the development of a therapeutic alliance which has been correlated with treatment adherence and improved quality of life in mental health contexts [4]. Healthcare organizations, especially primary and ambulatory care clinics, are increasingly adopting social needs screening and linkages to community-based and social service assistance programs to address social needs as a part of their core services. These programs typically involve baseline screening for social risks such as food insecurity, housing instability, lack of transportation, and utilities support with referral to programs that can help address identified needs. Referrals often link patients to programs outside a healthcare facility such as food pantries, utilities shutoff protections, or housing assistance programs, though in some settings they refer to assistance programs on-site, such as clinic-based food pantries or medical-legal partnerships. A recent paper described patient experiences with a screening and referral program, which provided key insights into how interactions with these programs impact patients both in terms of appropriateness of resource referrals and the establishment of a caring, therapeutic alliance [5]. However, that paper was unable to explore in depth the barriers those patients face when trying to connect to community resources.

We know that patients experience considerable barriers to accessing health-related services. For example, the American Hospital Association has laid out barriers to ensuring access to quality healthcare in vulnerable communities, including “limited federal funding, restrictive federal regulations, and a lack of collaboration and buy-in from community stakeholders” and indicates that “the most important resource may be hospital-community partnerships” [6]. Furthermore, disconnected relationships have been identified and described between clinical-community partnerships [7], and collaborative clinical-community partnerships are required to address patient social needs [8]. More work needs to be conducted to identify and describe barriers to accessing social needs-related services, especially when those needs are identified in a healthcare setting. To fill this gap and help to strengthen clinical-community partnerships, this paper is a deep dive into patient-reported barriers to accessing and using the social needs-related resources to which they are referred by a program embedded in a safety net primary care clinic. This analysis is based on over 100 in-depth interviews with individuals in Northern California who participated in a program embedded in a county run primary care clinic to help address their social needs. We propose a framework for understanding how policies and systems intersect with an individual’s personal life circumstances based on the findings from these in-depth interviews. These intersecting barriers create a complex web that combine in ways that compound patients’ challenges to getting their social needs met. Better understanding the type and range of barriers individuals face opens the door for better assessment of these barriers and more targeted support to help overcome those barriers. These findings also highlight the need for advocacy to reduce the systems barriers, especially the stigma that is faced by people who struggle with a variety of health and social issues and seeking assistance. These insights will help guide the design of policies and programs that are better able to address barriers patients experience when trying to seek assistance for social needs.

## Methods

Interviewees participated in an intervention that was a collaboration between Contra Costa Health System (CCHS) and the nonprofit organization Health Leads. CCHS is located in Contra Costa County in northern California, which has a total population of 1.03 million people [9]. The composition of the county’s population is 22.9% Hispanic individuals, 13.8% Asian/Pacific Islander and 9.0% African American; Hispanic individuals are the fastest growing ethnic group in the county [9]. West County Health Center (WCHC) is one of 11 health clinics run by CCHS to serve as safety net clinics for the Contra Costa County’s most vulnerable residents. In 2018, WCHC provided more than 140,000 patient visits to the community across a variety of services including primary care, women’s health and specialty services. The intervention pilot embedded trained volunteer patient advocates in a large primary care clinic to help patients access community resources [10].

The Health Leads volunteer advocate model is well-documented [3, 5, 11]. Patients are referred to advocates based on responses to a paper screening tool developed by Health Leads to assess social needs [12]. Advocates conducted phone or in-person needs assessments and helped patients access resources. After initial encounters, advocates were expected to follow-up via phone, text, email or in person to determine if patients successfully accessed resources, needed help addressing barriers, and had additional needs. We conducted purposive sampling based on a set of characteristics we wanted to make sure we captured, implemented a stratified sampling plan, and included quotas based on two characteristics: 1) 50% of sample with > 1 successfully met needs, and 2) 30% Spanish speaking, defined as patient’s preferred language. We also prioritized three additional characteristics: 1) date of case closure, prioritizing more recently served patients to reduce recall bias, 2) number of social needs met, ensuring inclusion of patients with multiple needs, and 3) gender. Recruitment letters were sent starting with individuals who had the most recent experiences with the program in order to reduce recall bias. We had both addresses and phone numbers so we able to follow up with individuals even if they were not currently housed and had not received the letter. We followed informed consent protocols to ensure that participants understood the project and voluntarily consented to participate. A full description of our sampling strategy, recruitment, data collection and analysis procedures for the interview data has been published previously [5].

Selection criteria included: 1) enrolled in an intervention that is designed to screen for essential social needs and connect individuals to services between June 2014–April 2016, 2) had at least one closed need during that time interval (indicating that the advocate had completed their work with the patient, and either successfully or not successfully met the identified needs). Successfully met needs indicate that the patient reported successfully accessing resources in their community. We excluded patients who did not speak English or Spanish and who were under 18 years of age. Recruitment was designed to include respondents with key demographics similar to those in the Health Leads program [13].

The interview guide was a 12-item guide designed to gather qualitative information from respondents about their experience working with advocates, including barriers experienced while trying to access resources in their communities, and if their needs were resolved (Appendix A). Semi-structured interviews were conducted via phone between July 1 and September 30, 2016 and averaged 15–30 min. Patients received $30 incentives by mail. Full description of data analysis has been described previously [5]. Interviews were coded using a thematic analysis approach. Coders developed an initial code list based on themes surfacing during review. All coders coded one transcript using the draft code list and compared their work. Codes were added, revised and clarified, repeating until all agreed the list was comprehensive. Each team member drafted sections of a master coding memo that was used to help summarize findings. Data were managed in Atlas.ti.

## Results

We sent recruitment letters to 275 patients, resulting in interviews with 102 individuals, for a 37% response rate. Our population was 62% female (Table 1). Thirty-eight percent (38%) were between 50 and 65 years, and 35% were 30–49 years old. Per recruitment goals, 70% of the interviews were conducted in English and 30% were conducted in Spanish. A full description of the recruited and interviewed sample has been previously published [5].

**Table 1:**
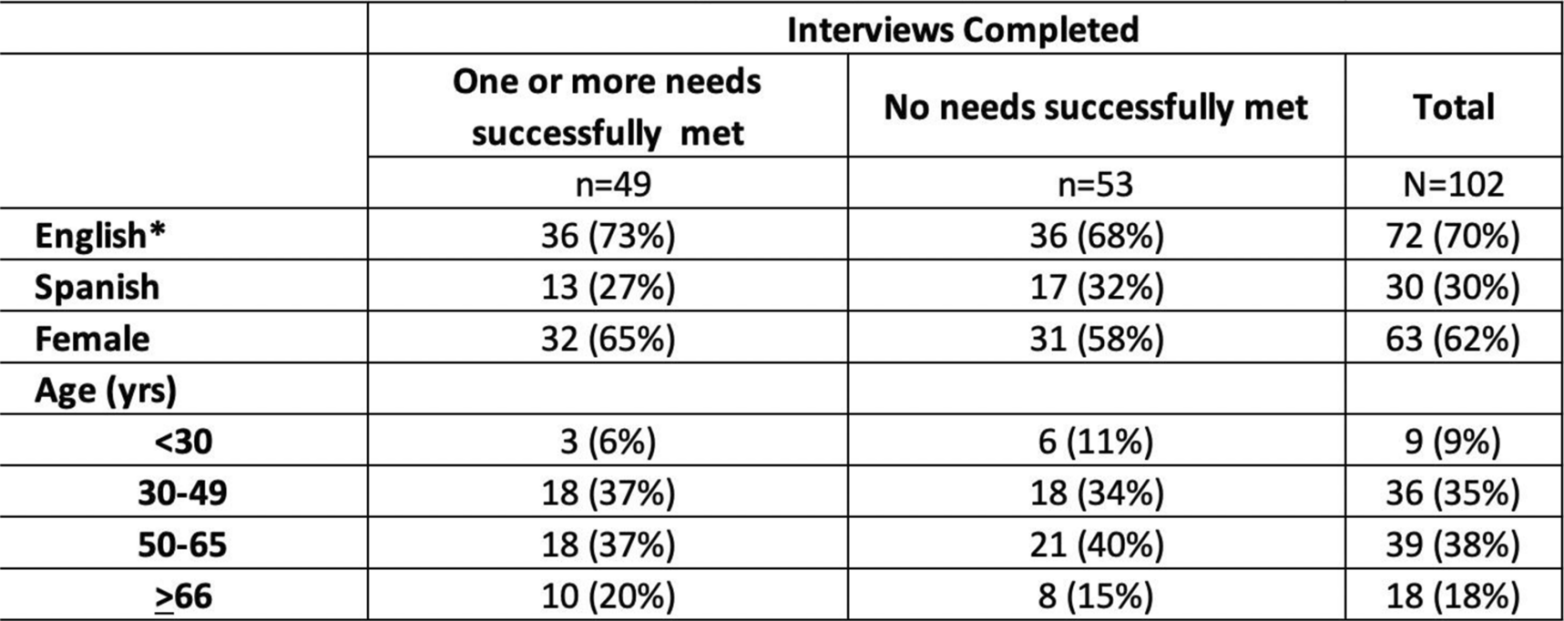
Characteristics of interviewed individuals (*n* = 102)

*“English speaker” may include someone with a preferred language other than Spanish and English who indicated at intake that they also speak English.

One overarching finding was that barriers clustered around two key domains: 1) systems-level barriers that were linked to the inequitable distribution of and access to resources, and 2) personal (individual)-level barriers that focused on unique limitations experienced by each patient who engaged with the Health Leads program and impacted the way that they accessed services in their communities. While these barriers often overlapped or manifested in similar outcomes, this distinction was key because the systems barriers were not things that individuals could control or overcome through their own initiative or by increasing individual capacity.

### Systems-level barriers

Systems-level barriers were commonly cited as the reason patients did not access the services.

Table 2 lists themes described by patients along with individual quotes illustrating each theme, including the following:

**Table 2:** Systems-level barriers to accessing resources, themes with supporting quotes

Theme	***Supporting Quotes***
**Limited availability of necessary resource**	*They know their clients need housing and all they basically got is like the counselors do the same thing there.They go online, they look what’s out there and they give their clients a stack of paper to call these numbers.But you call these numbers and there’s really literally nothing available, there really isn’t.People call and say “I’m looking for some housing” and they say what kind of income do you have?Oh, I don’t have any income, or people got Social Security, I mean what do people expect?You know what I mean?They do have shelters, if people are really that hard up for a place to stay there are shelters out there.There’s the Richmond shelter and a couple other ones that provide a bed and a couple meals and even a place to shower, come down to it. Male, English, No success*
	*Housing was a little bit of a disappointment.But I don’t think it was the fault of them, I think it’s the fault of the Bay Area. Male, English, No success*
	*Housing is very tricky...I had to opt for some sort of temporary, like a summer situation where I’m renting right now.But I haven’t really figured out the housing from that list.I did go and apply to get on a list for like disabled housing, but that didn’t really do much, because the lady did tell me “our list is pretty long and we only have how many units.”She recommended applying for a lot of places and then getting on as many waiting lists as possible, but it seemed like it was a long time, you know?Yeah, I didn’t get housing through there.It was mainly at a certain point I just realized I’ve got to do something, and I opted for like a summer sublet situation where I’m just renting a room for the summer, and then I’m not sure. Female, English, No success*
**Limited accessibility of necessary resource**	*Every time I called them nobody ever answered the phone so I had no idea when I could go up there.She didn’t give me any times to go up there, like if they have open pantries or whatever.There was no like specification.So nobody ever answered the phone there so I was just like whatever.I didn’t really call back, because I don’t think I have any other pantries that are really close to me.I’m not sure, but I mean now I could really use it because I don’t have food stamps anymore. Female, English, No success*
	*I don’t get around that well and the one [food pantry] I go to is really pretty far for me to walk.My only transportation is either my feet or a bicycle or public transportation out here.That’s not convenient to these places because unfortunately I have to walk someplace to get a bus to take it there. Male, English, No success*
	*For us it’s really expensive because we do not know Martinez and there’s no one that can take us, so we had to take the train to McArthur and from there at McArthur to Concord and from Concord to Martinez, like that. And we spent $56 on the fare. Female, Spanish, No success*
**Strict program criteria**	*They was saying I had too much money and I went to the Salvation Army in Richmond.They said I had too much money in the household.And then when I tried to explain the situation and whatever it was, they didn’t even want to go through all of that, so I just said forget it. Female, English, No success*
	*I don’t qualify for food stamps, I don’t qualify for SSI, I’m in a loophole where I don’t qualify for anything but I don’t get nothing...I can’t work.I have a seizure disorder, I’m 40 years old.I have a seizure disorder that’s very bad, that keeps me from working.I have a memory disorder.I can’t drive, I can’t be by myself.I stay busy 24/7 and I’m on a lot of medication.But because of my age, it’s hard for me to get help. Female, English, No success*
**Complicated application processes**	*It’s like you go to a place and you got to wait and then like you say, wait in line, and you got to fill out this paperwork and come back next week and see if you qualify.You got to play their little game with them...They say you got to wait for them to see if they accept you and approve it before they turn around and help you out. Male, English, No success*
	*The only thing, I’m going through that list now, the Social Security, I’m finished with that.I haven’t turned in the application yet though, because I went online to see what else they needed.There’s more information I got to put on there for the doctor’s part.But they were very helpful.It’s just a long process....Not just that, but also some places won’t tell you they’re accepting applications, you have to actually walk in.Because I had to walk into one that was down in Richmond so I’ve got to walk into the other ones too, to make sure.They don’t tell you anything.You have a list and you call them, but they probably want you to just come there. Female, English, No success*
**Stigma associated with necessary resource**	*No, I did not check the Albany pool, although a classmate of mine at Contra Costa College, she said, “Oh yeah, at the Albany pool they have a special group for low income people.”A group?It’s not like they plug you into a regular swimming class and no one knows about your low income except the clerk in the office.You know? (laughs) Female, English, Success*
	*And it does sound like a good amount of money, but no, my children said no. “No, mom, maybe other people who have a greater need than you can receive it.” For that reason, I did not do it. Because my children are supporting my husband and I with that. Female, Spanish, Success*
**Lack of fluency in English/access to interpreter**	*Particularly English is my second language and I’m not comfortable to ask any questions about anyone. Female, English, No success*
	*Sometimes in clinic or in the beginning they ask me if I need translator. Sometimes the translator, when they speak, I can’t understand well what they’re saying in French. I say sometimes okay, I can’t understand. I try to understand, to talk to the person. Because their French is sometimes too difficult for me to understand. Female, English, Success*
**Immigration status and legal policies**	*And I went to the module that’s in the middle of the hospital and they contacted me there and asked me for my social and I told them that no, I was an undocumented person. At the end they told me they could not make ther service available to me because it was for people who had documents. Female, Spanish, No success*
	*So I told them that and they told me that I should go to a lawyer, that maybe, that because the whole I time I was working with a social that the government could give me help for unemployment or for retirement. And I tell you, I was with the unión for 10 years, for 21 years. But it’s very difficult until you have papers, maybe that help will come later… Male, Spanish, Success*

### Limited availability

For a number of common social needs there were often not enough resources available. For example, housing is a common need that was often unavailable, especially in the Bay Area in Northern California, due to the limited options for safe and affordable housing.

### Limited accessibility

Services were often difficult to access due to either distance or challenges getting through via phone or online. Individuals who were employed described difficulty accessing services outside of work hours. For individuals with transportation or mobility challenges, they described difficulty physically accessing services either because they could not get the transportation they needed to get to the service, or once there, could not gain access to the building due to mobility challenges.

*I don't get around that well and the one [food pantry] I go to is really pretty far for me to walk. My only transportation is either my feet or a bicycle or public transportation out here. That's not convenient to these places because unfortunately I have to walk someplace to get a bus to take it there.* Male, English, No success

### Strict program criteria

Ineligibility for the service or resource because of income, health status, insurance status, lack of necessary documentation or identification, citizenship status, age or another program participation was a common barrier that patients encountered. An example of this is becoming ineligible for Supplemental Nutrition Assistance Program (SNAP) upon receiving disability benefits. Or, for instance, if an individual’s current income did not meet program requirements, they were ineligible for services. Respondents described feeling caught in a hole where they could not afford basic needs, for instance, but were also ineligible from getting assistance to help with those needs. Prior to June 1, 2019, this was a very common barrier for many patients. Fortunately, starting June 1, 2019 in CA, people receiving Supplemental Security Income (SSI) can also apply for and receive CalFresh (SNAP) without impacting their SSI amount.

### Complicated application processes

SNAP is a strong example of this, because it often includes a lengthy application, difficult questions, and multiple steps to complete.

### Stigma

Perceptions of stigma associated with the necessary resource often kept respondents from accessing the resource. Respondents described feeling embarrassed to use some services due to the fact that those who access particular programs may be seen as low-income, incapable, or handicapped.

### Lack of fluency in English and access to an interpreter

Language difficulties impacted how individuals could access resources; some respondents described feeling uncomfortable asking questions in English, particularly if English was their second language. Others described lack of adequate interpreter or translators which impacted how they could access services:

*Sometimes in clinic or in the beginning they ask me if I need translator. Sometimes the translator, when they speak, I can't understand well what they're saying in French. I say sometimes okay, I can't understand. I try to understand, to talk to the person. Because their French is sometimes too difficult for me to understand.* Female, English, Success

### Immigration status and legal policies

This, coupled with lack of trust in institutions, impacted how individuals could access services. Services that require proof of citizenship are not only inaccessible to undocumented individuals but discouraged use of services by citizens with undocumented family members.

### Personal-level barriers

Personal-level barriers were described by patients as preventing them from accessing the services or support they requested or were offered by Health Leads. The most common themes are illustrated in quotes shown in Table 3, including the following:

**Table 3:** Personal-level barriers to accessing services, themes with supporting quotes

Theme	***Supporting Quotes***
**Physical and mental health challenges**	*I still need help, not as far as the papers, but I need help because right now what’s going on with me - I guess my disability has run out and so now, because I’m an injured worker, I’ve already started filing for the Social Security Disability, but I need to be finding me a job that can work around my injury.Because my injury is with my hand, I lost like 48% of the use of my hands and my arms, and I was just retrained for the Dragon program, I need help with finding resources as far as the type of job I can work that doesn’t go against my restrictions. Female, English, No success*
	*I really was not expecting anything because I was not right in my head.Female, English, Success*
	*It’s still the same, still going through it.I haven’t been able to work because of the arthritis I got so it really messes me up.I try to do little things here and there to try and keep my body active, but it hurts doing those things.I can only do certain little things I can, and then after a minute it just hurts me and everything else like that, so I can only limit myself to what I can do.I’ve been talking to a lot of people, employers, and they’re like “we can’t hire you in your condition.When you get better, come back when you get better and everything else.”But it’s been two years and it hasn’t been better yet, so it’s kind of frustrating. Male, English, Success*
**Social isolation, and lack of social support**	*Oh, she listened and she gave me information after I told her about my case and stuff, and she said “oh, you probably qualify for this and that,” so she gave me the information and she mailed me some papers in the mail.But like I said, it’s hard for me to get help and I live here by myself plus my friend being at work, it’s mainly through the week that I need the services and stuff.And I can’t make it after work, they be closed. Female, English, No success*
	*I don’t have a driver’s license....I have to wait when my daughter in law or my son, when they are available, but everybody is working. Saturday as you know, the county offices are closed, but the food pantries are open, so that is good. Female, English, Success*
**Lack of technology resources and computer illiteracy**	*I get food stamps, but I don’t get the money assistance because I don’t want to be one of those guys that sucks off the government, I want to find a job, I want to make my own way through this life, you know what I mean.. She gave me the Web address, but unfortunately because I’m so low on money, I’m just barely able to afford my phone so I get the lowest possible data plan possible so I run out of high speed Internet real quick, and a lot of the Web pages I try to load for doing applications just seem to halt for some reason... Male, English, No success*
	*I checked out some of those ones, because I’m computer illiterate and some places where I could go to, just to sit down, just to get peace of mind and kind of learn how to get on the internet and stuff like that.That was very helpful for me because I had never been online before.Someone was trying to teach me a long time, I want to say 2011, but as they were teaching me, they fell off so I just didn’t even pursue it.Because when I would ask people, they would tell me, and I’m more was a hands on person when it comes to stuff like that.By her giving me that information I was able to go to the library and just get online and kind of breeze through places for rent and who was renting and what’s not, jobs and stuff like that.And it was really cool to get on the Internet, to go to the library. Female, English, no success*
**Financial limitations**	*I am a senior on very little income.I did use that some years back, 15, I don’t know, and it worked for me.But the YMCA is a business and they’re in the business of making money.So I did call them when I was looking for swimming lessons and they said they no longer do that.Because I have to have a membership, there’s no waiver for the membership which in my budget is beyond my ability. Female, English, Success*
	*The only thing that didn’t work out with me was that I wanted to become a citizen, and she gave me the phone number for that and kept calling all those numbers, but there was nothing, I didn’t get anything.I didn’t have the kind of money, it’s about a thousand dollars to get to the citizenship things.I was trying to get some help there, and then I tried to call that girl, but she told me she was going to a different place or something, and I didn’t take any more help from there. Female, English, Success*
**Competing Priorities**	*Because when you’re dealing with emotional problems with doing practical things, if there’s one more person leaning on you to get it done, it’s not helpful for somebody like me.I don’t know what the answer is to getting them to get it done - extreme consequences like losing Medicaid.Obviously I was willing to lose food stamps for two months rather than turn around and get the paperwork done. Female, English, No success*
	*Well, my car broke down so I’m waiting to get my car fixed, because I haven’t been able to do anything lately.My catalytic converter went out, and it’s really expensive and so I’m just waiting to get enough money.It’s sitting right in my driveway, stuck right in front of my front door.So I’m trying to get up the money to get that fixed.And then I can go out and do all the things that I want to do. Male, English, No success*
	*I remember telling them about some of my bills, they were past due and I wanted to see if they could help me, especially the water bill at that time...I knew I was going to need them, definitely, but not at that point.I was in the hospital probably every day.Blood work, blood pressure check up, MRI, so I didn’t have time really to contact them or go to the clinic and look for them... Male, English, No success*

### Physical and mental health challenges

Individuals often described the ways that limitations imposed by health conditions posed a considerable barrier to getting their social needs met. This includes coping with symptoms of mental illness that made it difficult to engage with service organizations, chronic medical conditions like arthritis that causes severe pain, or acute medical conditions like injuries which made it difficult to find and access services to meet social needs.

### Social isolation and lack of social support

Respondents described how living alone and/or lacking support from friends and family hampered their ability to access services. This barrier often interacted with physical health challenges since individuals with pain or limited mobility were dependent on others to accompany them places in order to access services. In addition, those without a driver’s license described feeling dependent on others to be able to access services:

*I don't have a driver's license....I have to wait when my daughter in law or my son, when they are available, but everybody is working. Saturday as you know, the county offices are closed, but the food pantries are open, so that is good.* Female, English, Success

### Lack of technology resources and computer illiteracy

Respondents without technology resources or who are computer illiterate described challenges to accessing services that were only accessible via the Internet.

### Financial limitations

Respondents described having inadequate finances for the requested resource. For respondents with little income, they described challenges to accessing services that had financial costs associated with them, including issues requiring legal aid, or physical activity programs to help with chronic or acute pain or injuries.

### Competing priorities

Respondents were often dealing with multiple personal- and systems-level barriers simultaneously and respondents reported that their need was not a high priority in comparison to other events or tasks that they were addressing.

Sometimes this was coupled with a lack of confidence that the resource would meet needs given past lived experiences or pressing new concerns. These comments also included the perception that the individual costs associated (time, effort) were too high to justify the anticipated reward, even when the reward was significant.

Our findings also indicated that there is a strong interrelationship between the systems and personal barriers patients encountered when presented with Health Leads resource referrals or attempting to access them. For example, a person with mobility issues (a personal barrier) might cite the distance of services from their home and limited public transportation as a challenge (a systems barrier):

*“Well, my car broke down so I'm waiting to get my car fixed, because I haven't been able to do anything lately. My catalytic converter went out, and it's really expensive and so I'm just waiting to get enough money. It's sitting right in my driveway, stuck right in front of my front door. So I'm trying to get up the money to get that fixed. And then I can go out and do all the things that I want to do.” (Male, English, No success).*

Other respondents describe health-related barriers that speak to the complexity of everyday life that can be difficult to navigate without personal resources like social support or financial resources:

*“I remember telling them about some of my bills, they were past due and I wanted to see if they could help me, especially the water bill at that time...I knew I was going to need them, definitely, but not at that point. I was in the hospital probably every day. Blood work, blood pressure check up, MRI, so I didn't have time really to contact them or go to the clinic and look for them...” Male, English, No success*

Some of the respondents possessed behavioral, cognitive or developmental disabilities, and some described experiencing memory problems, social anxiety, and obsessive-compulsive disorders within the interview which made it difficult to work to access services:

*“Because when you're dealing with emotional problems with doing practical things, if there's one more person leaning on you to get it done, it's not helpful for somebody like me. I don't know what the answer is to getting them to get it done - extreme consequences like losing Medicaid. Obviously I was willing to lose food stamps for two months rather than turn around and get the paperwork done.” (Female, English, No success)*

For some, the need to access services to address social risks represented a loss of dignity. For others, stigmatized feelings were combined with a feeling of not wanting to “burden” the healthcare system or to take away resources from “more needy” patients/people. This was a key theme particularly identified among some Spanish speakers:

*“And it does sound like a good amount of money, but no, my children said no. “No, mom, maybe other people who have a greater need than you can receive it.” For that reason, I did not do it. Because my children are supporting my husband and I with that.” Female, Spanish, Success*

Sometimes interviewees lacked the capacity to articulate why they were unable to access resources. In other cases, respondents expressed confusion about the rules and regulations governing their benefits or who can access benefits from the organizations they were referred to, often large bureaucracies such as local or federal housing programs, county social services agencies, and public utilities.

## Discussion

Our study found that there were two interwoven yet distinct domains of barriers. Systems barriers were pervasive and included adequacy and accessibility issues along with perceived issues of stigma and complex application and eligibility processes. Personal barriers included a host of problems taking priority over or complicating the ability for patients to access services to meet their needs including medical concerns, social isolation or lack of social support, fear of discrimination, or lack of technology literacy. Personal-level barriers compounded their difficulties accessing resources. Some resources—especially those related to legal aid, citizenship, and adult education —appear to have costs to patients, putting them out of reach for patients with little or no income. Patients reported physical health limitations or mental health conditions like depression that made meeting daily obligations challenging. Many of the interviewed patients often expressed the barriers they faced in ways that sounded like issues of prioritization: doing what was required to access resources—making phone calls, completing applications, going to new agencies—simply do not rise to the top of the list of things important to patients’ or their families’ well-being. Respondents described that demands of daily living are so challenging that mounting any extra effort seemed too difficult, if not impossible. Another interpretation of some of the patients’ descriptions speak to matters of self-efficacy or confidence. Patients were not certain that they could even do what the advocates advised, or if they did succeed, they expressed a lack of confidence that the resource would address their needs. While these problems may be relatively uncommon in the population overall, among the target audience for Health Leads they appear frequently.

With this in mind, a key finding from our analysis indicates that any program staff or volunteers hoping to engage patients struggling with personal barriers may benefit from additional training, triaging clients into special protocols for goal setting, action planning and monitoring. Patients may need more time with an advocate to overcome these barriers in order to successfully approach and resolve the resource need.

Our findings contribute substantially to reports from other studies assessing barriers to receiving clinical or social services. Similar to our findings, those studies report cultural, socioeconomic, and legal barriers [14, 15] as well as attitudinal barriers such as not wanting to involve outsiders in personal problems, not seeing the need for services, availability of services, language barriers or concerns about cultural appropriateness [16]. Other studies identifying barriers to accessing health and social services have reported high rates of self-reported access issues, and have identified that those reporting access issues were more likely to be socially and economically vulnerable or suffering from mental health conditions indicating that those who have the hardest time accessing services may be the most in need [17, 18]. Studies identifying how barriers to clinical and social services can be reduced use targeted social support interventions and specific navigation models to promote patient-centered health improvements [19] as well as strategies to evaluate local resources to develop partnerships and disease prevention interventions [20, 21]. Most studies raise the need to better understand “the relationship between perceived barriers to accessing services and dissatisfaction with services” [16] in order to promote health equity in our communities.

These results contribute substantially to the literature by describing the relationships between system- and personal-level barriers to accessing services among individuals experiencing social risks and engaged in an intervention to access services in their communities. Respondents describe intersecting systemic and personal barriers that combine in ways that compound patients’ challenges to getting their social needs met; this includes both a picture of the inequitable distribution of and access to social services and a profile of the limitations created by individual life histories. These findings also provide a framework for addressing both systemic and personal-level barriers to accessing social services in communicatees in northern California. Overall, our results speak to the need for structural changes to improve adequacy, availability, and accessibility of social needs resources. More intentional investments are needed to better understand how to address the deep institutional, systemic, and personal issues that respondents describe. Based on the findings above, we recommend the following:

- More investment in organizations focusing on building hospital/clinic-community partnerships in scalable ways. We need organizations committed to strengthen partnerships focused on providing whole person care including assessing the quality and quantity of local resources and investing in local networks and relationships. Some examples include organizations like 2–1-1 systems help promote the collaboration between public utility companies, housing organizations, food banks, and legal services (among others) [22], or Community Connect, Contra Costa County’s Whole Person Care Pilot program under the 1115 Waiver [23]. This program utilizes a multidisciplinary team that includes case managers from both Health and Social Services agencies to provide extended social needs case management services which includes counselling services through a relationship-based model that is trauma-informed and employs motivational-interviewing techniques to develop client prioritized care plans to connect individuals to resources in their communities and tracks patient-level outcomes over time to understand impact on utilization and overall health.
- Better understanding of the competing priorities that make accessing services a low or negligent priority for some respondents. Results indicate that typically fewer than half of those who screen positive for social risk factors are interested in receiving assistance to help address identified risks [24, 25]. More analysis should be conducted to better understand if other priorities are more urgent in an individual’s current circumstances, or if there is a combination of barriers, perspectives, and experiences at play. Projects addressing this specifically are currently being led by researchers at UCSF, with support from the Robert Wood Johnson Foundation and Kaiser Permanente [26, 27].
- Improved clinical incentives enabling program staff to engage with patients struggling with systems- or personal-level barriers to accessing social services. In this analysis, we describe a high-touch program that screens individuals for social needs, connects those who screen positive to services in their communities, and a system for longitudinal follow-up to better understand resolution of reported social needs. These programs take time and investment to operate successfully, and this can be difficult, or nearly impossible, in already overloaded clinical environments. Optimizing navigation programs (like the program that respondents in this paper participated in) requires continued investment in program staff and the time and space needed to build trusting relationships. Creating economic incentives for prevention and upstream investments in health encourage health plans and integrated health networks to invest in social interventions [28] which will enable program staff to improve care delivery for those struggling with social needs.

### Limitations

Interviews, although asked in as supportive a manner as possible, were posed by strangers with no relationship to the patient. Asking patients to explain their internal thought processes in this context may make it hard for patients to be forthcoming; there may be embarrassment about not following through, a sentiment that patients expressed by blaming themselves for not making the effort or following their advocate’s suggestions. In addition, this study was limited to English and Spanish speakers. We were only able to speak with individuals who spoke English or Spanish, therefore we do not know if individuals who don’t speak either of these languages have more or different barriers. Other limitations include recruitment at a single health clinic in northern California, and a sample limited to participants who were reachable by telephone and consented to study participation. However, since the intervention occurred primarily over the phone and the vast majority of those who participated in the program had phones, it would have excluded very few program participants, if any.

## Conclusions

Interviewers asked patients about anything that prevented them from accessing services or anything that the Health Leads advocates could have done to remove those barriers. What emerged included both a picture of the inequitable distribution of and access to social services and a profile of the limitations created by individual life histories. While the program these individuals participated in made efforts to help address the social needs of individuals struggling with health issues, it was difficult to overcome the complex interrelationship of personal and systemic barriers. Understanding the range of barriers individuals face when trying to access resources for social needs calls for the need for more targeted support to help overcome those barriers. These findings also highlight the need for advocacy to address the systems barriers, especially the stigma that is faced by people who struggle with a variety of health and social issues, and investment in incentives to strengthen relationships between healthcare organizations and social service agencies.

## Data Availability

The data that support the findings of this study are available from Kaiser Permanente but restrictions apply to the availability of these data, which were used under license for the current study, and so are not publicly available. Data are however available from the authors upon reasonable request and with permission of Kaiser Permanente.

## Patient Interview Guide

### Health Leads Patient Interview Guide

1. Can you describe to me how you were introduced to the people in the blue shirts-the Health Leads advocates?
  **PROBES:**
  - Do you remember filling out any forms? What were your thoughts when you were asked to filling out the needs assessment form?
  - What did your doctor or any one on your doctor’s team tell you about the Health Leads program?
  - What was your reaction to being offered this service?
2. I’d like to know how you felt about the person or people you talked with, the advocate/person in the blue shirt at the clinic, or the person who called you to offer program services.
  **PROBES:**

- How well do you feel they listened to you?
- Do you feel they understood your life circumstances/what is going on in your life (for example what you could reasonably do and afford, or other barriers or challenges you faced)?
- Overall, how comfortable were you talking with this person? What about them made you feel more or less comfortable?

3. Can you describe your first conversation with the Health Leads advocate?
  **PROBES:**
  - Did you talk to them when you were at the clinic? On the phone?
  - When you did talk with them was it at a convenient time?
  - What did you talk about with them?
  - Were the services the Health Leads person offered what you expected after talking with the doctor or health care team? (or below)
  - Did you have the time you needed to fully describe your needs?
  - Was there anything else they did or said during this first conversation that you found particularly helpful?
  - What, if anything, surprised you about the help they were offering?
4. [LOWER PRIORITY: ONLY ASK IF NOT COVERED IN QUESTIONS 3 **AND** YOU ARE NOT MORE THAN 20 MINUTES INTO THE INTERVIEW] What specific information did they give you about resources to contact?
  **PROBES:**
  - How did you feel about the resources they suggested? Did they make sense to you?
  - Had you heard of any of these resources before you worked with the Health Leads person>
  - Were the resources they offered what you expected? How were they different? (too far away, too expensive, not eligible for them, etc.)?
  - Were there other resources you needed help connecting with?
  - Did the help they provided give you any other ideas (or skills) that helped you address the issue you needed help with?
5. Did you try to contact the resources they suggested? Why or why not?
6. <If they attempted to contact> Can you tell me the story of/about what happened when you tried to contact the resource?
  **PROBES:**

- <probe as needed to fully understand their experience with the resource>
- Who contacted the resource?
- What happened when you got ahold of the resource/service?
- How were you treated?
- Did they offer the services you thought they were going to offer?

7. What are the main barriers you came up against when trying to either contact or use the resources you were referred to?
8. Were there things that the Health Leads program could have done to make contacting the resource easier?
9. Did the Health Leads program follow up with you to see if you were able to get the resources you needed? If yes, can you describe that follow up call?
  **PROBES:**
  - Did you actually speak to someone?
  - What types of questions did they ask you?
  - Did they give you any additional information?
  - How useful did you feel this follow up call was?
  - How much follow up do you want from a program like Health Leads? Would you have liked them to follow up more? Less?
  - What time of day did they call you? Was this a convenient time for you? If not, what would have been more convenient?
10. What is going on now with regard to your <name issue, i.e. housing situation, access to food >?
11. What has changed as a result of working with the HL program?
  **PROBES:**
  - What, if anything, has changed with regard to your health as the result of working with the HL program (or that you would link to getting the resource you were referred to)?
  - Did the HL program give you any information or coaching that helped you feel better about your health? If so, what?
  - Did the HL program give you the confidence to reach out to other resources on your own? If so, can you tell me about that?
  - How, if at all, did your experience with the HL program change how you feel about your doctor and/or your doctor’s team?
  - What, if any, other changes occurred for you or your family as a result of working with the Health Leads program?
12. Overall, how would you describe your feelings about the help you received from the HL program?
  **PROBES:**
  - How satisfied were you?
  - Would you use this service again? Why or why not?
  - Did you have any concerns about the services provided by the HL program?
  - How did you feel amount the amount of support they provided?
    i. Was it too much? Too little?
    ii. If too much, what would you have preferred they didn’t do?
    iii. If too little, what more could they have done to support you in getting resources or services?
  - How did you feel about receiving this service at your clinic/doctor’s office?
  - What did you like best about Health Leads program?
  - What could the Health Leads person or program have done better?

## Abbreviations

COVID-19: The name of the disease caused by the novel coronavirus SARS-CoV2
LDL: Low-density lipoprotein
CCHS: Contra Costa Health System
WCHC: West County Health Center
SNAP: Supplemental Nutrition Assistance Program
SSI: Supplemental Security Income
UCSF: University of California San Francisco
